# Transforming Traditional Korean Medicine hospital EHRs into the OMOP common Data Model: methodology and implications

**DOI:** 10.1186/s12911-026-03418-z

**Published:** 2026-03-16

**Authors:** Man Young Park, Jaeuk U. Kim, SunMi Choi, Youngheum Yoon, Byung-Kwan Seo, Sangkwan Lee

**Affiliations:** 1 Digital Health Research Division, Korea Institute of Oriental Medicine, Daejeon, Republic of Korea; 2 Department of Data Science for Korean Medicine, Korea Institute of Oriental Medicine, Daejeon, Republic of Korea; 3 Big Data Center for Korean Medicine, National Institute for Korean Medicine Development, Seoul, Republic of Korea; 4 Department of Acupuncture & Moxibustion, College of Korean Medicine, Kyung Hee University Hospital at Gangdong, Kyung Hee University, Seoul, Republic of Korea; 5 Department of Internal Medicine and Neuroscience, College of Korean Medicine, Wonkwang University, 460 Iksan-daero, Sin-dong, Iksan, Jeollabuk-do 54538 Republic of Korea; 6 School of Korean Convergence Medical Science, University of Science and Technology, Daejeon, Republic of Korea

**Keywords:** Data standardization, Electronic health records, Evidence-based medicine, OMOP Common Data Model, Traditional Korean Medicine

## Abstract

**Background:**

Standardizing data from Traditional Korean Medicine (TKM) is essential for enhancing interoperability with international real-world data infrastructures, such as multi-institutional OMOP-CDM databases within the OHDSI network, multinational claims databases, and large-scale clinical data repositories, thereby enabling evidence-based research. This study aimed to convert electronic health records (EHRs) from a TKM hospital into the Observational Medical Outcomes Partnership (OMOP) Common Data Model (CDM).

**Methods:**

We transformed the EHR data from Wonkwang University Gwangju Korean Medicine Hospital into the OMOP CDM format. TKM-specific diagnoses, procedures, and medications were mapped to standard vocabularies, and new concept codes were developed when no standard terms were available. Beyond a direct application of conventional CDM transformation procedures, we established explicit mapping principles and introduced structural extensions-such as the KIOM terminology system-to accommodate pattern identification, complex herbal prescriptions, and detailed acupuncture techniques that could not be represented within existing vocabularies. An Extract, Transform, Load (ETL) process was conducted, and data quality was evaluated using the ACHILLES tool.

**Results:**

The converted dataset, named the Wonkwang Traditional Korean Medicine (WKTKM) database, included records from 88,449 patients. It comprised more than 4 million condition records, approximately 10 million drug prescriptions, and over 10 million procedure records. Most laboratory results and medications were successfully mapped to existing standard concepts, while TKM-specific concepts were integrated using a newly developed terminology system called the KIOM codes.

**Conclusion:**

This proof-of-concept study demonstrates the technical feasibility of deterministic, rule-based transformation of EHR data from a TKM hospital into the OMOP CDM. By incorporating methodological extensions-including the temporary creation of KIOM terminology and the development of generic mapping rules for TKM-specific diagnostic and therapeutic elements-this study illustrates a practical baseline pathway for harmonizing traditional medicine data. While the WKTKM database establishes an important methodological foundation, extensive cross-institutional validation and centralized terminology governance are necessary prerequisites before such standardized datasets can be reliably deployed for large-scale, generalizable international collaborative research.

**Supplementary Information:**

The online version contains supplementary material available at 10.1186/s12911-026-03418-z.

## Introduction

The use of medical big data is bringing significant changes to both medical research and clinical practice [1, 2]. In particular, the widespread and increasingly common adoption of Electronic Health Records (EHRs) across various healthcare institutions has made it possible to utilize large volumes of systematically collected data from real-world clinical settings as primary research sources. This has contributed to improved understanding of patient care and health outcomes [3, 4]. Such real-world data are now widely used in areas such as clinical research, healthcare quality improvement, and health policy development [5].

However, differences in data structures and coding systems across institutions pose challenges for conducting multi-institutional or international comparative studies [6, 7]. These discrepancies hinder the integration and comparability of healthcare data between systems, thereby limiting large-scale observational research and evidence generation in real clinical environments [8].

To address these challenges, the Observational Health Data Sciences and Informatics (OHDSI) consortium proposed the Observational Medical Outcomes Partnership Common Data Model (OMOP-CDM) [9]. The OMOP-CDM enables the transformation of heterogeneous medical data into a standardized structure [10], supporting the development of large distributed research networks and collaborative studies [11]. Numerous studies have reported successful examples of data standardization and analysis using the OMOP-CDM [12–15].

Traditional Korean Medicine (TKM), with its long history and unique theoretical framework, is an essential part of the healthcare system in Korea [16]. However, the diagnostic systems, prescriptions, and treatment methods used in TKM differ considerably from Western medicine–based international standards, making data standardization challenging. This gap arises from fundamental differences in medical philosophy, diagnostic logic, and treatment representation. TKM is primarily based on pattern identification (e.g., Qi deficiency, blood stasis) and holistic syndromes rather than disease entities alone, whereas the OMOP Common Data Model (CDM) is structured around disease-centered classifications derived from modern biomedical paradigms, such as ICD and SNOMED CT. In addition, TKM prescriptions frequently consist of multi-herbal decoctions in which therapeutic meaning emerges from the combination and proportional composition of ingredients, while RxNorm is designed to represent single active pharmaceutical ingredients or standardized pharmaceutical products. Acupuncture treatments further complicate standardization, as existing OMOP vocabularies lack structured representations for acupoints, meridian systems, and needling techniques.

As a result, direct one-to-one mapping between TKM concepts and existing international vocabularies is often infeasible, necessitating structural reinterpretation and terminology extension. Consequently, the standardization and international sharing of TKM data remain in the early stages, which limits the advancement of TKM research and its global academic visibility. As a result, the standardization and international sharing of TKM data remain in the early stages, which limits the advancement of TKM research and its global academic visibility [17].

There is a growing need to convert and integrate TKM data in accordance with international standards. This approach provides a foundation for systematizing TKM research, advancing evidence-based Korean medicine, and enabling global collaboration, highlighting the necessity of adopting the OMOP-CDM framework.

This study aims to describe the process and outcomes of converting electronic medical record data from a TKM hospital into the OMOP-CDM. Specifically, we present: (1) the methods used to map TKM-specific diagnostic, prescription, and procedure codes to OMOP-CDM standard vocabularies; (2) details of the Extract, Transform, Load (ETL) process; and (3) the results of the quality assessment of the converted dataset.

## Methods

### Research setting

This study utilized electronic medical record (EMR) data from Wonkwang University Gwangju Oriental Medicine Hospital, located in Gwangju, South Korea. Established in 1994, the 300-bed tertiary oriental hospital offers various oriental medical services, including herbal medicine, acupuncture, and moxibustion.

The hospital’s EMR system stored the data in a proprietary structure using a unique coding system for diagnosis, herbal medicine, and procedure. The dataset included patient demographics, oriental medicine diagnosis (pattern identification), herbal medicine prescriptions, acupuncture, and other clinical information collected from January 1, 2012 to December 31, 2022. This study was conducted in accordance with the Declaration of Helsinki and was approved by the Institutional Review Board of Wonkwang University (approval number: WKIRB2024-12). Clinical trial number: not applicable.

### OMOP common data model

**Fig. 1:**
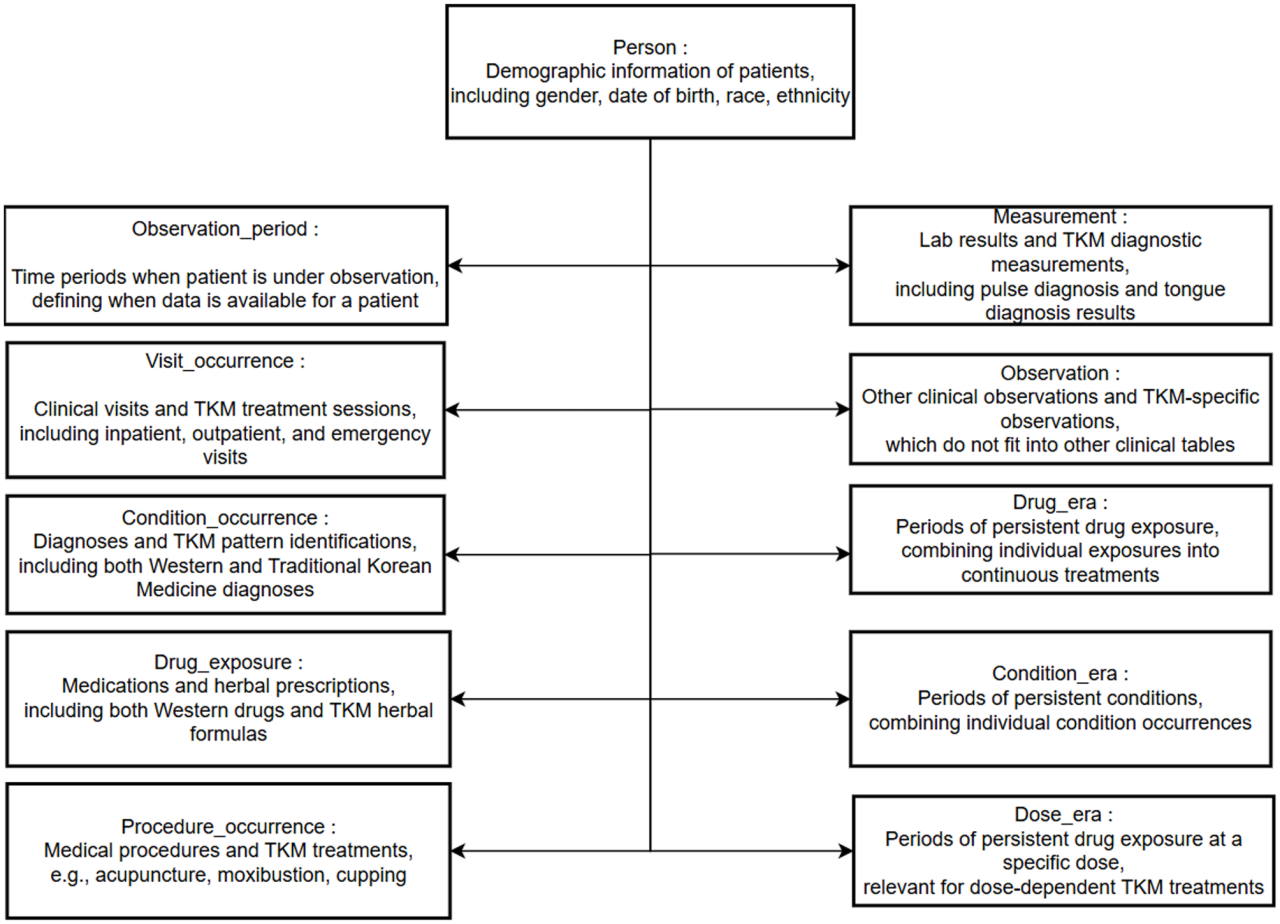
Main OMOP CDM v5.3 Tables for Traditional Korean Medicine Data. This schema illustrates the key OMOP CDM domains used to represent electronic health records from TKM hospitals, including standard clinical domains (e.g., drug exposure, procedures) and TKM-specific elements such as pulse diagnosis, tongue diagnosis, acupuncture, and herbal prescriptions

In this study, we used OMOP CDM version 5.3, a relational database schema designed to standardize various medical data sources. The model consists of four main areas: standardized clinical data, healthcare system data, health economy data, and metadata (Fig. 1).

### Code mapping process

The conversion of electronic medical records from Wonkwang University Gwangju Korean Medicine Hospital to OMOP CDM required systematic mapping of source codes to standard terminologies. Key clinical data categories included laboratory examinations, acupuncture procedures, herbal medicine ingredients, and herbal medicine formulations. We adopted a comprehensive mapping strategy involving multiple steps. First, we identified and selected key standard terminology systems used in the OMOP CDM framework, such as SNOMED-CT, LOINC, and RxNorm. Then, we meticulously defined the OMOP CDM domains suitable for each code category and established mapping criteria based on priorities. This foundation allowed us to select representative sample codes from each category and perform real-world mapping tasks.

For the mapping process, we mainly used the ATHENA website (athena.ohdsi.org) provided by OHDSI. We set the mapping criteria for each category considering the appropriate domain, vocabulary, and standard conceptual state. Laboratory testing, for example, prioritized standard concepts by using measurement domains and selecting LOINC as the main vocabulary, but also considered classification concepts or non-standard concepts if necessary.

Recognizing the unique characteristics of Traditional Korean Medicine (TKM), we encountered a substantial number of domain-specific concepts that could not be directly mapped to existing international standard vocabularies such as SNOMED CT or RxNorm. To address this limitation, we developed an institutional terminology extension named the “KIOM terminology”, derived from the Korea Institute of Oriental Medicine (KIOM), where this research was conducted. The KIOM terminology is not an external standard ontology but a locally developed controlled vocabulary created specifically for this study to encode TKM-specific diagnostic patterns, herbal prescriptions, and acupuncture procedures that lacked adequate representation in existing standard systems. Each KIOM concept was assigned a unique concept_id within the OMOP CDM framework and was registered as an independent vocabulary with structured metadata, including English concept names and Korean-language synonyms. New concepts were systematically generated following predefined semantic rules described in Supplementary Material S1. These rules included domain classification, hierarchical structuring, concept naming conventions, and consistency checks to ensure semantic coherence and internal validity.

Beyond the technical requirements of code conversion, this mapping approach introduced methodological refinements specifically tailored to the characteristics of Traditional Korean Medicine. The TKM domain includes diagnostic constructs (e.g., pattern identification such as Qi deficiency and blood stasis), complex multi-herb formulations, and detailed acupuncture techniques that do not have direct counterparts in existing international vocabularies such as SNOMED CT or RxNorm. To address these structural disparities, we established explicit mapping principles and redefined conceptual hierarchies to determine appropriate CDM domains for TKM concepts. In cases where the semantic gap was unavoidable, we designed the KIOM terminology system as a structured extension aligned with OMOP conventions to preserve clinical meaning while enabling interoperability. This methodological framework provides a reproducible decision-making process that can guide the standardization of traditional medicine data beyond routine code conversion.

### ETL (Extract, Transform, Load) Process

The ETL process has been carefully designed and implemented to accurately convert traditional Korean medicine (TKM) data from Wonkwang University Gwangju Oriental Medicine Hospital into OMOP CDM format. This process was executed by combining R (version 4.1.2) for data manipulation and analysis and SQL for database operation, and the final data was loaded into a PostgreSQL (version 13.4) database structured according to the OMOP CDM schema. The complete ETL code is publicly available on GitHub (https://github.com/ManYoungPark/KCDM_OMOP).

### WKTKM database construction

The dataset transformed into OMOP was designated as the WKTKM (Wonkwang Traditional Korean Medicine) database. We constructed this database by applying code mapping and ETL processes to the original EMR data while adhering to the OMOP CDM structure and preserving the unique characteristics of TKM data.

### Data quality control

To verify the quality of data in the ETL process, we utilized ACHILLES developed by OHDSI. ACHILLES is an open-source analysis software that operates on OMOP Common Data Model (CDM) versions 4 and 5, serving as a tool to assist in quality assessment and visualization of all data used in OMOP. ACHILLES computes summary statistics for database content within each local environment and generates interactive dashboards for information in each table. Notably, ACHILLES includes a unique data quality check feature called Achilles Heel, which plays a crucial role in Data Quality Management (DQM). We identified issues arising from Achilles Heel in our dataset, systematically resolved the problems, and employed a method of re-executing the ETL process when necessary.

## Results

### WKTKM database characteristics

The WKTKM database included data from 88,449 patients. Table 1 presents the demographic and clinical characteristics of the WKTKM database population.

**Table 1:** Demographic and clinical characteristics of the WKTKM database population

Characteristic	Value
Total patients	88,449
Age (mean ± SD)	51.7 ± 19.2
Gender (female)	48,511 (54.8%)
Outpatient visits	1,048,575
Inpatient visits	23,085
Observation length (days, mean ± SD)	1.6 ± 6.0
Unique conditions	681
Conditions per patient (mean)^*^	41.4
Unique herbal medicines	2299
Prescriptions per patient (mean) ^*^	112.7
Unique lab tests	19
Lab results per patient (mean) ^*^	30.9
Unique procedures	573
Procedures per patient (mean) ^*^	118

^*^The average number of conditions, prescriptions, lab results and procedures per patient was calculated by first determining the count of conditions for each individual patient and then computing the mean across all patients

### Code mapping results

To evaluate mapping performance with clinical relevance, we adopted a domain-stratified sampling strategy. Specifically, we selected the top 20 laboratory tests, top 10 Western medicine drugs, and top 60 traditional Korean medicine (TKM) prescriptions based on usage frequency. Because laboratory tests and Western medicine drugs are already well covered by existing OMOP standard vocabularies, a smaller representative set was sufficient for baseline validation. In contrast, TKM prescriptions were expected to have substantial semantic mismatch with existing terminologies; therefore, a larger number of commonly used prescriptions was intentionally evaluated. Among laboratory tests, 100% of the selected items were successfully mapped to standard OMOP concepts. For Western medicine drugs, 100% were directly mapped to RxNorm or standard drug concepts. In contrast, only 35% of TKM prescriptions could be fully represented using existing standard vocabularies without terminology extension. After applying the KIOM terminology system, 100% of the selected TKM prescriptions were successfully represented within the CDM structure.

For laboratory test codes, most could be directly mapped to LOINC or SNOMED CT concepts. For instance, ‘XH002003’ (Hgb) was mapped to OMOP concept_id ‘3000963’ (Hemoglobin [Mass/volume] in Blood). However, for some tests, accurate mapping was challenging due to unclear specimen or unit information.

Acupuncture codes were mostly mapped to the general concept of ‘Acupuncture’ (concept_id: 4260518). However, this had limitations in fully reflecting the detailed information of TKM-specific acupuncture procedures. For example, code ‘4001200413’ (Acupuncture on Baihui and Quchi points using Five Phase method for head, neck, and upper limb) was simply mapped to ‘Acupuncture’, resulting in loss of information about the procedure site and method. To address this, we created new mapping codes. Table 2 shows examples of mapping acupuncture codes to OMOP CDM concepts.

**Table 2:** Mapping examples of acupuncture codes to OMOP CDM concepts

Category	Original Code	Original Code Name	concept id / new concept id	concept name / new concept name
Acupuncture	4,001,200,413	Acupoint acupuncture (head and neck + upper limb, Five Phase method)	4,260,518	Acupuncture
Acupuncture	4,001,203	Acupoint acupuncture (head and neck + upper limb)	4,260,518	Acupuncture
Acupuncture	40,060	Intra-articular acupuncture	4,260,518	Acupuncture
Acupuncture	40,080	Penetration acupuncture method	4,260,518	Acupuncture
Acupuncture	40,091	Electroacupuncture	44,790,076	Electroacupuncture

Note: Standard mappings to OMOP concepts (e.g., concept_id: 4260518, ‘Acupuncture’) often lacked procedural detail. New concept mappings were created to preserve TKM-specific information

For herbal medicine ingredient codes, most could be mapped to RxNorm or SNOMED CT concepts. For example, ‘DAGC’ (Glycyrrhiza uralensis) was mapped to concept_id ‘1353048’ (licorice) (Table 3). However, for some herbal ingredients, finding accurate English names or scientific names was challenging.

**Table 3:** Mapping examples of herbal medicine ingredient codes to OMOP CDM concepts

Code	Korean Name	Concept id	Concept name
DAGC	감초	1,353,048	licorice
DADG	당귀	42,898,329	Angelica gigas root extract
DAJP	진피	35,198,020	citrus unshiu peel
DABBR	백복령	40,220,850	Wolfiporia extensa whole extract
DACGG	천궁	42,898,897	Cnidium officinale root extract

Note: The ‘code’ column represents the actual codes used in the hospital’s electronic health record system

Herbal decoction codes were mostly impossible to map to existing OMOP concepts. To resolve this, we defined a new terminology system called ‘KIOM’ and assigned new concept_ids starting from 900,000,001. For instance, ‘JTS0001’ (Gamidaebotang decoction [excellent empirical prescription]) was mapped to the newly created concept_id ‘900000021’ (Gamidaebotang decoction) (Table 4).

**Table 4:** Mapping examples of herbal decoction codes to OMOP CDM concepts

Code	Code name	New concept id	New concept name
JTS0001	개별처방[탕약]	900,000,020	Other decoction (personal prescription)
JTS0001	가미대보탕(우수경험방)[탕약]	900,000,021	Gamidaebotang decoction
DC341	지해소청룡탕(DC)(포)	900,000,022	Jihaesocheonglyongtang granules
JTS0001	순기활혈탕(청강의감)[탕약]	900,000,023	Sungihwalhyeoltang decoction (cheongganguigam)
JTS0001	소풍탕가미[탕약]	900,000,024	Sopungtang-gami decoction

Note: The ‘code’ column represents the actual codes used in the hospital’s electronic health record system

Through this mapping process, we were able to convert the data to fit the OMOP CDM structure while maximally maintaining the unique characteristics of TKM data. However, there were limitations in fully preserving information, especially for TKM-specific concepts such as detailed acupuncture procedure information or complex herbal decoction prescriptions. Table 5 shows comprehensive mapping examples from each category.

**Table 5:** Comprehensive mapping examples of various korean medicine codes to OMOP CDM concepts

Category	Original Code	Original Code Name	concept id / new concept id	concept name / new concept name
Lab Test	XH002003	Hgb	3,000,963	Hemoglobin [Mass/volume] in Blood
Acupuncture	4,001,200,413	경혈침술(두경부+상지부,오행침법)	900,000,001	Acupunture on baeghoe, gogji by Ohaeng mathod (head and neck, upper extremity)
Herbal Medicine	DAGC	감초	1,353,048	licorice
Herbal Decoction	JTS0001	개별처방[탕약]	900,000,020	Other decoction (personal prescription)
Western Medicine	137801ATB	Colchine(Colchicine 0.6 mg)	1,101,556	colchicine 0.6 MG Oral Tablet

Note: The ' Original Code’ column represents the actual codes used in the hospital’s electronic health record system

### ETL process results

The ETL process resulted in the successful conversion of the original EMR data to the OMOP CDM format. The process yielded 4,015,386 condition occurrences, 9,968,003 drug exposures, and 10,472,161 procedure occurrences in the WKTKM database. Although the ETL process was technically successful across all OMOP CDM tables, semantic completeness varied by data category. Laboratory tests and Western medicine drug codes were mapped with near-complete accuracy using LOINC and RxNorm, respectively. In contrast, substantial limitations were observed in TKM-specific domains. Most acupuncture procedures, compound herbal decoctions, and pattern diagnoses could not be represented using existing international vocabularies. These gaps did not reflect ETL failure but rather structural incompatibility between TKM concepts and OMOP terminologies. To address this, new KIOM concepts were introduced for domains where direct mapping was infeasible.

### Data quality control results

Upon initial application of ACHILLES Heel to the transformed dataset, a total of 42 errors and 15 warnings were identified. These issues exhibited distinct patterns commonly observed in OMOP CDM conversion projects and could be categorized into four major groups.

First, domain-vocabulary inconsistencies were frequently detected, wherein mapped concepts were assigned to CDM tables incompatible with their semantic domain (e.g., procedure concepts erroneously placed in the OBSERVATION table). These discrepancies were systematically resolved through comprehensive review of domain assignments and subsequent re-mapping to domain-appropriate standard concepts. Second, unmapped or incompletely mapped source codes resulted in records with concept_id = 0, predominantly affecting TKM-specific clinical entities including traditional diagnoses, herbal prescriptions, and acupuncture methods. This limitation was addressed by augmenting the KIOM terminology system with newly defined concepts and re-executing the ETL process. Third, foreign key constraint violations were identified, including references to non-existent person_id or visit_occurrence_id values. Root cause analysis traced these errors to incomplete join operations between source demographic and encounter tables during initial ETL development. Resolution required restructuring the ETL logic to enforce strict referential integrity throughout the transformation pipeline.

Fourth, laboratory-related warnings emerged from inconsistent unit representations, clinically implausible value ranges, and missing essential attributes such as specimen information. Manual review of flagged records informed revision of transformation rules to standardize unit expressions and exclude semantically invalid entries.

Through iterative correction cycles and repeated validation using ACHILLES, all critical errors were eliminated and warning counts were substantially reduced, thereby confirming both the structural validity and analytical readiness of the WKTKM dataset for subsequent research applications.

### Data visualization

**Fig. 2:**
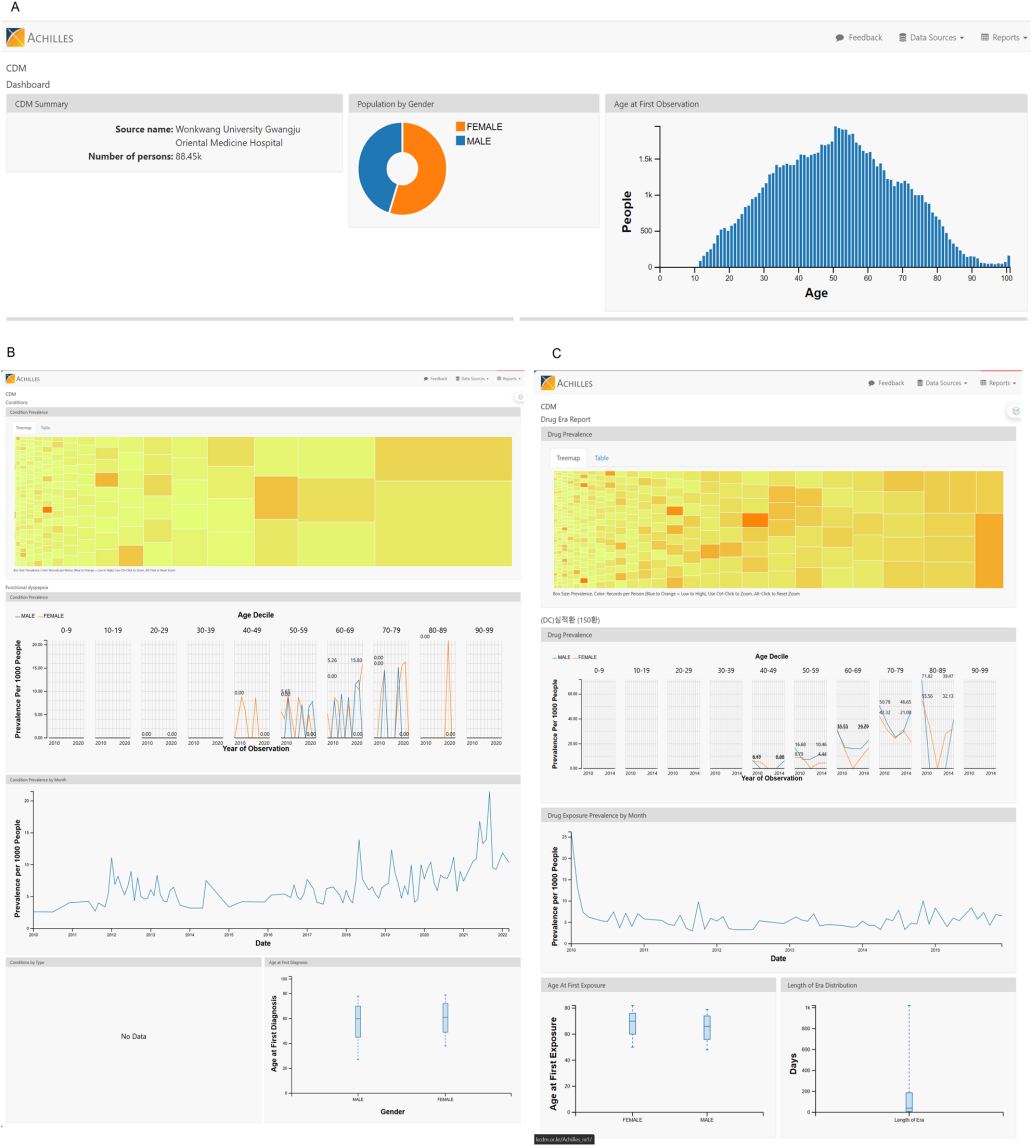
WKTKM database visualized using ACHILLES (Automated Characterization of Health Information at Large-scale Longitudinal Evidence Systems). (**A**) Basic information about the population in the database is shown in the dashboard tab. (**B**, **C**) The prevalence and number of records per person are shown using the size and color of the boxes in the tree maps at the tops of the following tabs: Conditions, Condition Eras, Observations, Drug Eras, Drug Exposures, Procedures, and Visits. Trends and related information for the selected box in the tree map at each tab are visualized below the tree map. (**B**) Data for Functional Dyspepsia is shown, which is included in the Conditions tab of the OMOP common data model. (**C**) Data for Shim-Jeok-Hwan, a traditional Korean herbal formula, is displayed. The graph shows the trend of its usage over time

The ACHILLES web interface for the WKTKM dataset is available at [http://kcdm.or.kr/Achilles_re1/#/CDM/dashboard] (Fig. 2). This interactive platform allows users to explore and visualize various aspects of the converted dataset, providing valuable insights into the characteristics and quality of the WKTKM database.3.1.

## Discussion

This study succeeded in converting the electronic medical records of Wonkwang University Gwangju Oriental Medicine Hospital into OMOP CDM, which is the first study of international standardization of oriental medicine data.

### Methodological contribution

While the present study is based on data from a single institution, its primary contribution is not the dataset itself but the methodological framework developed for representing Traditional Korean Medicine (TKM) within the OMOP Common Data Model. Unlike conventional CDM conversion studies that focus on Western medicine–based EHRs, this work addresses fundamental structural mismatches between TKM and existing international vocabularies by introducing explicit mapping principles, terminology extension strategies, and domain reinterpretation rules. The KIOM terminology system, together with the mapping logic described in this manuscript, provides a transferable blueprint that other institutions can adopt independently. In this regard, the value of this work lies in establishing a reproducible methodology rather than reporting a one-time institutional implementation.

### Challenges

Particularly noteworthy was the difficulty of mapping the diagnostic concepts and treatments unique to oriental medicine into the standard vocabulary system of OMOP CDM. It was impossible to map oriental medical diseases such as *Qi deficiency* (氣虛) or *blood stasis* (瘀血) on a one-to-one basis to the disease classification system of Western medicine. To solve this problem, a new term system called KIOM code was developed. By developing and using this term system, we tried to secure compatibility with international data standards while preserving the unique theoretical framework of oriental medicine.

The conversion process presented several challenges that required solutions, most notably the mapping of TKM-specific concepts, particularly pattern diagnoses and herbal prescriptions, to standard vocabularies. To address this, we adopted an approach of creating new concepts by extending SNOMED-CT and RxNorm, then linking these to existing terminology systems. For instance, pattern diagnoses were added as sub-concepts within the SNOMED-CT hierarchy, while herbal prescriptions were decomposed into individual herb components before mapping to RxNorm. Additionally, for some unstructured data, we developed algorithms utilizing natural language processing techniques to extract structured information from text, thereby improving data completeness and accuracy.

### Dataset & practical implications

The WKTKM database established through this study contains the records of 88,449 patients, which is the first large-scale dataset converted to OMOP CDM in the field of oriental medicine. The results of this study have significant significance for TKM research and clinical practice.

The results of this study have significant implications for TKM research and clinical practice.

First, this conversion work established a foundation for including TKM data in international observational research networks. TKM researchers can now utilize OHDSI network analysis tools, enabling multi-center comparative effectiveness research and large-scale observational studies. Second, data standardization greatly enhanced the reusability and interoperability of TKM data, creating pathways for single-institution data to be shared with researchers worldwide and expanding potential applications in various future studies. Third, beyond technical standardization, the WKTKM database provides a practical foundation for clinical and translational research in traditional medicine. By transforming heterogeneous TKM records into a standardized format, this framework enables comparative effectiveness studies between traditional Korean medicine and Western medicine, safety surveillance of herbal prescriptions, and longitudinal outcome analysis across large patient populations.

Furthermore, integration into the OMOP ecosystem allows researchers to leverage OHDSI analytical tools for cohort identification, treatment pathway analysis, and outcome modeling, thereby facilitating study designs that were previously infeasible in traditional medicine research. Importantly, this standardized infrastructure supports evidence generation in integrative medicine settings, where hybrid treatment strategies combining herbal medicine and Western pharmaceuticals can be systematically evaluated using real-world clinical data.

### KIOM long-term strategy

Beyond the immediate mapping requirements, the KIOM terminology system was designed as a long-term infrastructure for sustainable standardization of TKM concepts. The terminology is maintained through a governance framework that includes hierarchical classification structures (diagnosis–procedure–prescription), versioning protocols (e.g., v1.0, v1.1), and periodic expert review by a committee of TKM specialists. To ensure future interoperability with international standards, we have established integration strategies for SNOMED CT and RxNorm. For SNOMED CT, KIOM diagnostic and procedural concepts will be mapped to corresponding “finding” and “procedure” hierarchies through cross-reference tables. For RxNorm, complex herbal prescriptions will be decomposed into ingredient-level components, enabling linkage with standard drug codes. Additionally, we are preparing a documentation framework to formally propose TKM-specific concepts for inclusion in SNOMED CT International. These efforts position the KIOM terminology as a sustainable and interoperable component within the global terminology ecosystem, supporting future multi-institutional and international research collaborations.

## Limitations

While this study created an important precedent bridging traditional medicine and modern medical data science, several challenges remain. First, the data were obtained from a single tertiary traditional Korean medicine (TKM) hospital, which may limit the generalizability of the findings to other institutions or healthcare settings. Second, although the OMOP CDM conversion process successfully standardized most clinical information, several TKM-specific domains-such as detailed acupuncture procedures, pulse and tongue diagnoses, and complex herbal formulations-could not be fully represented within the existing OMOP vocabulary structure. Consequently, our decision to use generic mapping concepts conceptually prioritized structural feasibility and cross-institutional interoperability in this initial phase. However, this approach inherently risks information loss regarding site-specific granular terminology. Future multi-institutional studies, incorporating comparative gap analyses, are essential to develop more granular mapping strategies that balance data harmony with maximal information preservation. Third, the new KIOM terminology developed in this study has not yet been externally validated or officially integrated into international vocabularies such as SNOMED CT or RxNorm, requiring further refinement and consensus. The current terminology governance framework, while sufficient for this proof-of-concept study, remains an internal institutional standard; transitioning this to a multi-institutional committee will be necessary for sustainable, standardized version histories and expert consensus protocols. Fourth, it is important to clarify that our ETL framework underwent structural validation to ensure deterministic rule-based transformations executed without errors, rather than formal clinical chart reviews assessing the medical appropriateness of those underlying mapping rules across diverse settings. Such clinical validation requires broader governance and multi-site deployment. Furthermore, our sampling strategy explicitly concentrated on high-risk domains like TKM prescriptions where semantic mismatch was most anticipated, rather than achieving a perfectly symmetrical sampling across all clinical domains. Fifth, the dataset cannot be shared publicly due to institutional data use restrictions, which may limit the reproducibility of our findings by external researchers. Finally, this study focused primarily on the technical feasibility of data transformation rather than on downstream clinical or analytical applications, which should be addressed in future multi-institutional projects.

## Future directions

Despite these limitations, this research holds important significance in establishing a foundation for traditional medicine to demonstrate its value within the paradigm of modern evidence-based medicine. Future research directions should include large-scale CDM conversion projects incorporating multi-institutional TKM data. Standardized datasets built through such efforts would enable various empirical studies, including comparative effectiveness research of specific herbal prescriptions or clinical utility assessments of integrated Korean-Western medicine treatments.

Based on our research experience, we propose the following directions for international standardization of TKM data. First, systematic collaboration is needed to officially incorporate TKM concepts into international standard terminology systems like SNOMED-CT and RxNorm [18–20]. China and Japan are already working to include their traditional medicine terminologies in international standards, and Korea should actively participate in these efforts. Second, CDM extension models capable of accommodating TKM-specific diagnostic methods such as pulse and tongue diagnosis need to be developed. Third, consensus on data standardization should be formed within the Korean medicine community, and inter-institutional collaborative networks should be established.

## Conclusion

In conclusion, this proof-of-concept study demonstrated the technical feasibility of deterministic, rule-based transformation of traditional Korean medicine hospital electronic health records into the OMOP Common Data Model. The process introduced temporary generic mapping strategies and the internal KIOM terminology system to structurally represent TKM-specific concepts within the CDM framework. While this work establishes an important methodological foundation, it is critical to recognize that achieving true clinical interoperability and robust multi-institutional data harmony requires substantial subsequent efforts. Extensive cross-institutional validation, formal comparative gap analyses, and the establishment of a centralized terminology governance committee are necessary prerequisites before these standardized datasets can be reliably deployed for large-scale, generalizable clinical research and safety surveillance within global research infrastructures.

## Supplementary Information

Below is the link to the electronic supplementary material.

Supplementary Material 1

## Data Availability

The dataset generated and analyzed during the current study cannot be shared publicly due to institutional restrictions and data use regulations. The data were obtained under a data use agreement with Wonkwang University Gwangju Korean Medicine Hospital and are therefore not available for public access. However, the complete conceptual mapping framework and representative mapping rules are provided in Supplementary Material S1 to enable independent reproduction of the methodology.

## Abbreviations

ACHILLES: Automated Characterization of Health Information at Large-scale Longitudinal Evidence Systems
CDM: Common Data Model
EHR: Electronic Health Record
EMR: Electronic Medical Record
IRB: Institutional Review Board
KIOM: Korea Institute of Oriental Medicine (and the KIOM terminology for TKM-specific concepts)
LOINC: Logical Observation Identifiers Names and Codes
OHDSI: Observational Health Data Sciences and Informatics
OMOP: Observational Medical Outcomes Partnership
RxNorm: Prescription Normalized Names
SNOMED CT: Systematized Nomenclature of Medicine-Clinical Terms
TKM: Traditional Korean Medicine
WKTKM: Wonkwang Traditional Korean Medicine database
