# Supplementary material for: Transforming Traditional Korean Medicine hospital EHRs into the OMOP common Data Model: methodology and implications
Source: BMC Med Inform Decis Mak. 2026 Mar 16;26:139. doi: 10.1186/s12911-026-03418-z (PMC13104197; doi:10.1186/s12911-026-03418-z)
Supplement: Supplementary file 1 — Supplementary Material 1 [file 12911_2026_3418_MOESM1_ESM.docx]

Supplementary Material S1. Detailed Mapping Principles for Traditional Korean Medicine (TKM) Terminologies to OMOP CDM

This supplementary document provides detailed narrative descriptions and extended tables supporting the code-mapping methodology described in the main manuscript. Because much of the Traditional Korean Medicine (TKM) terminology lacks direct equivalents in OMOP standard vocabularies, explicit mapping principles and sample mapping results are provided to improve transparency and reproducibility.

**S1. Mapping Principles by Terminology Category**

**1. Laboratory Test Codes**

- **Domain:** Measurement
- **Vocabulary priority:** LOINC → SNOMED CT → Others
- **Standard concept priority:** Standard (S) → Classification (C) → Non-standard (NS)
- **Notes:**
  - Multi-concept mapping not allowed (1 test = 1 row).
  - When an exact match is unavailable, higher-level parent concepts were used.
  - Additional information such as specimen type and unit is required for future refinement.

**2. Acupuncture Procedure Codes**

- **Domain:** Procedure
- **Vocabulary priority:** SNOMED CT → CPT4 → ICD9Proc
- **Standard concept priority:** S → C → NS
- **Notes:**
  - Almost all acupuncture procedures collapse to SNOMED’s *Acupuncture* concept (4260518).
  - To preserve specificity (e.g., acupoint location, method such as Five-Phase or Tui-Na style), **new KIOM concepts** were generated.
  - Body-site modifiers in OMOP (modifier_concept_id) were insufficient for traditional acupoint representation.

**3. Herbal Ingredient Codes**

- **Domain:** Drug
- **Vocabulary priority:** RxNorm → SNOMED → Others
- **Notes:**
  - Common herbs map well to RxNorm Extension.
  - Some herbs have multiple botanical species sharing the same Korean name; mapping is based on the most widely accepted pharmacopeial botanical name.
  - Additional metadata (root/leaf/whole extract) improves precision.

**4. Herbal Decoction Codes**

- **Domain:** Drug
- **Vocabulary priority:** RxNorm (rarely available) → All vocabularies
- **Notes:**
  - No standard OMOP concepts exist for classic TKM decoctions.
  - Thus, new **KIOM decoction concepts** were created (concept_ids starting from 900000020).
  - Decoctions marked as “포(包)” were interpreted as granular preparations.

**5. Western Medicine Ingredient Codes**

- **Domain:** Drug
- **Vocabulary:** RxNorm
- **Concept class:** Clinical Drug preferred
- **Notes:**
  - Brand names in Korean EMR were removed during mapping (standard OMOP practice).
  - For combination drugs, multi-concept mapping was applied only when necessary.

# ****6. Creation of New KIOM Concepts****

A narrative summary of the KIOM concept creation process:

- Korean descriptions were translated into English; when translation was ambiguous, standardized Romanization was used.
- A new vocabulary record (“KIOM”) was added to the OMOP vocabulary table.
- Each newly created concept includes:
  - concept_id, concept_name, domain_id, vocabulary_id, concept_class_id, valid_start_date, and synonyms (including original Korean term).
- Example:
  - 900000001 – Acupuncture on baeghoe, gogji by Ohaeng method
  - Synonym: Acupuncture (head and neck + upper extremity, Ohaeng method)

**S2. Sample Mapping Tables**

| **local code** | **code_name** | **concept_id** | **concept_code** | **concept_name** | **concept_class_id** | **standard** | **validity** | **domain_id** | **vocabulary_id** |
| --- | --- | --- | --- | --- | --- | --- | --- | --- | --- |
| XH002003 | Hgb | 3000963 | 718-7 | Hemoglobin [Mass/volume] in Blood | Lab Test | Standard | Valid | Measurement | LOINC |
| XCP0031 | Creatinine | 3016723 | 2160-0 | Creatinine [Mass/volume] in Serum or Plasma | Lab Test | Standard | Valid | Measurement | LOINC |
| XH002001 | WBC | 3010813 | 26464-8 | Leukocytes [#/volume] in Blood | Lab Test | Standard | Valid | Measurement | LOINC |
| XH002002 | RBC | 3026361 | 26453-1 | Erythrocytes [#/volume] in Blood | Lab Test | Standard | Valid | Measurement | LOINC |
| XH002009 | PLT | 3007461 | 26515-7 | Platelets [#/volume] in Blood | Lab Test | Standard | Valid | Measurement | LOINC |
| XH002202 | Eos# | 3013115 | 26449-9 | Eosinophils [#/volume] in Blood | Lab Test | Standard | Valid | Measurement | LOINC |
| XH002303 | Baso# | 3006315 | 26444-0 | Basophils [#/volume] in Blood | Lab Test | Standard | Valid | Measurement | LOINC |
| XCP0023 | ALT (GPT) | 3006923 | 1742-6 | Alanine aminotransferase [Enzymatic activity/volume] in Serum or Plasma | Lab Test | Standard | Valid | Measurement | LOINC |
| XCP0022 | AST (GOT) | 3013721 | 1920-8 | Aspartate aminotransferase [Enzymatic activity/volume] in Serum or Plasma | Lab Test | Standard | Valid | Measurement | LOINC |
| XH002004 | Hct | 3009542 | 20570-8 | Hematocrit [Volume Fraction] of Blood | Lab Test | Standard | Valid | Measurement | LOINC |
| XH002007 | MCHC | 3003338 | 28540-3 | MCHC [Mass/volume] | Lab Test | Standard | Valid | Measurement | LOINC |
| XH002005 | MCV | 3024731 | 30428-7 | MCV [Entitic volume] | Lab Test | Standard | Valid | Measurement | LOINC |
| XH002006 | MCH | 3035941 | 28539-5 | MCH [Entitic mass] | Lab Test | Standard | Valid | Measurement | LOINC |
| XH002010 | MPV | 3001123 | 28542-9 | Platelet mean volume [Entitic volume] in Blood | Lab Test | Standard | Valid | Measurement | LOINC |
| XH002012 | PDW | 3039417 | 51631-0 | Platelet distribution width [Ratio] in Blood | Lab Test | Standard | Valid | Measurement | LOINC |
| XH002008 | RDW | 3002385 | 30385-9 | Erythrocyte distribution width [Ratio] | Lab Test | Standard | Valid | Measurement | LOINC |
| XH002307 | Eos% | 3006504 | 26450-7 | Eosinophils/100 leukocytes in Blood | Lab Test | Standard | Valid | Measurement | LOINC |
| XH002308 | Baso% | 3022096 | 30180-4 | Basophils/100 leukocytes in Blood | Lab Test | Standard | Valid | Measurement | LOINC |
| XH002201 | Lym% | 3002030 | 26478-8 | Lymphocytes/100 leukocytes in Blood | Lab Test | Standard | Valid | Measurement | LOINC |
| 4001200413 | Acupuncture (head, neck + upper extremity, Ohaeng method) (Baeghoe, Gogji) | 900000001 | KIOM1 | Acupunture on baeghoe, gogji by Ohaeng mathod (head and neck, upper extremity) | Procedure | Standard | Valid | Procedure | KIOM |
| 4001200413 | Acupuncture (head, neck + upper extremity, Ohaeng method) (Sangseong, Cheogtaeg) | 900000002 | KIOM2 | Acupunture on sangseong, cheogtaeg by Ohaeng mathod (head and neck, upper extremity) | Procedure | Standard | Valid | Procedure | KIOM |
| 4001203 | Acupuncture (head, neck + upper extremity) (Baeghoe, Gogji) | 900000003 | KIOM3 | Acupunture on baeghoe, gogji (head and neck, upper extremity) | Procedure | Standard | Valid | Procedure | KIOM |
| 4001203 | Acupuncture (head, neck + upper extremity) (Baeghoe, Habgog) | 900000004 | KIOM4 | Acupunture on baeghoe, habgog (head and neck, upper extremity) | Procedure | Standard | Valid | Procedure | KIOM |
| 4001203 | Acupuncture (head, neck + upper extremity) (Yepung, Habgog) | 900000005 | KIOM5 | Acupunture on yepung, habgog (head and neck, upper extremity) | Procedure | Standard | Valid | Procedure | KIOM |
| 4001203 | Acupuncture (head, neck + upper extremity) (Pungji, Yeolgyeol) | 900000006 | KIOM6 | Acupunture on pungji, yeolgyeol (head and neck, upper extremity) | Procedure | Standard | Valid | Procedure | KIOM |
| 4001210 | Acupuncture (upper + lower extremity) (Habgog, Jogsamli) | 900000007 | KIOM7 | Acupunture on habgog, jogsamli (upper and lower extremity) | Procedure | Standard | Valid | Procedure | KIOM |
| 40060 | Intra-articular acupuncture (Gyeon-u) | 900000008 | KIOM8 | Acupuncture on gyeon-u (intra-articular) | Procedure | Standard | Valid | Procedure | KIOM |
| 40060007 | Intra-articular acupuncture - additional (50%) (Gyeon-u) | 900000009 | KIOM9 | Additional acupuncture on gyeon-u (intra-articular) | Procedure | Standard | Valid | Procedure | KIOM |
| 40060007 | Intra-articular acupuncture - additional (50%) (Jungbong) | 900000010 | KIOM10 | Additional acupuncture on jungbong (intra-articular) | Procedure | Standard | Valid | Procedure | KIOM |
| 40120 | Bungu method acupuncture (ear, head, foot, hand, nose, wrist, skin) | 900000011 | KIOM11 | Acupunture by Bungu mathod (on ear, head, foot, hand, nose, wrist joint and skin) | Procedure | Standard | Valid | Procedure | KIOM |
| 40070007 | Intervertebral acupuncture - additional (50%) (Daechu) | 900000012 | KIOM12 | Additional acupuncture on daechu (Intervertebral) | Procedure | Standard | Valid | Procedure | KIOM |
| 40091 | Electroacupuncture | 44790076 | 2.24721E+14 | Electroacupuncture | Procedure | Standard | Valid | Procedure | SNOMED |
| 40080 | Tuja method acupuncture (Gonlyun, Taegye) | 900000013 | KIOM13 | Acupunture on gonlyun, taegye by Tuja mathod | Procedure | Standard | Valid | Procedure | KIOM |
| 40080 | Tuja method acupuncture (Naegwan, Oegwan) | 900000014 | KIOM14 | Acupunture on naegwan, oegwan by Tuja mathod | Procedure | Standard | Valid | Procedure | KIOM |
| 40080 | Tuja method acupuncture (Sam-eumgyo, Hyeonjong) | 900000015 | KIOM15 | Acupunture on sam-eumgyo, hyeonjong by Tuja mathod | Procedure | Standard | Valid | Procedure | KIOM |
| 40080 | Tuja method acupuncture (Imun, Cheong-gung) | 900000016 | KIOM16 | Acupunture on imun, cheong-gung by Tuja mathod | Procedure | Standard | Valid | Procedure | KIOM |
| 40080 | Tuja method acupuncture (Jichang, Hyeobgeo) | 900000017 | KIOM17 | Acupunture on jichang, hyeobgeo by Tuja mathod | Procedure | Standard | Valid | Procedure | KIOM |
| 40080 | Tuja method acupuncture (Taeyang, Solgog) | 900000018 | KIOM18 | Acupunture on taeyang, solgog by Tuja mathod | Procedure | Standard | Valid | Procedure | KIOM |
| DE008 | Hwangnyeon detoxification pharmacopuncture 1ml | 900000019 | KIOM19 | Hwangnyeon detoxification acupuncture 1ml | Procedure | Standard | Valid | Procedure | KIOM |
| DAGC | Licorice | 1353048 | 42769 | licorice | Ingredient | Standard | Valid | Drug | RxNorm |
| DADG | Angelica gigas root | 42898329 | 1307664 | Angelica gigas root extract | Ingredient | Standard | Valid | Drug | RxNorm |
| DAJP | Citrus unshiu peel | 35198020 | OMOP4819385 | citrus unshiu peel | Ingredient | Standard | Valid | Drug | RxNorm Extension |
| DABBR | Wolfiporia extensa | 40220850 | 1803878 | Wolfiporia extensa whole extract | Ingredient | Standard | Valid | Drug | RxNorm |
| DACGG | Cnidium officinale root | 42898897 | 1307974 | Cnidium officinale root extract | Ingredient | Standard | Valid | Drug | RxNorm |
| DASK | Ginger root | 1314928 | 125921 | ginger root | Ingredient | Standard | Valid | Drug | RxNorm |
| DAISM | Korean ginseng root | 19099102 | 260038 | Korean ginseng root | Ingredient | Standard | Valid | Drug | RxNorm |
| DABC | Atractylodes macrocephala root | 43525881 | 1372633 | Atractylodes macrocephala root extract | Ingredient | Standard | Valid | Drug | RxNorm |
| DABH | Pinellia ternata root | 42899896 | 1307663 | Pinellia ternata root extract | Ingredient | Standard | Valid | Drug | RxNorm |
| DABPW | Saposhnikovia divaricata root | 42899731 | 1305761 | Saposhnikovia divaricata root extract | Ingredient | Standard | Valid | Drug | RxNorm |
| DAGHL | Notopterygium incisum root | 42899614 | 1307976 | Notopterygium incisum root extract | Ingredient | Standard | Valid | Drug | RxNorm |
| DAOAK | Lindera aggregata root | 43526252 | 1420971 | Lindera aggregata root extract | Ingredient | Standard | Valid | Drug | RxNorm |
| DABJ | Angelica dahurica root | 42898328 | 1306167 | Angelica dahurica root extract | Ingredient | Standard | Valid | Drug | RxNorm |
| DAMH | Saussurea costus root | 42899744 | 1309463 | Saussurea costus root extract | Ingredient | Standard | Valid | Drug | RxNorm |
| DASJH | Rehmannia glutinosa root (steamed 9 times) | 42899772 | 1309220 | Rehmannia glutinosa root extract | Ingredient | Standard | Valid | Drug | RxNorm |
| DAMMD | Liriope muscari root | 45893001 | 1600741 | Liriope muscari root extract | Ingredient | Standard | Valid | Drug | RxNorm |
| DADJ | Ziziphus | 43532231 | 1426423 | Ziziphus extract | Ingredient | Standard | Valid | Drug | RxNorm |
| DAGGG | Platycodon grandiflorum root | 42899925 | 1307614 | Platycodon grandiflorum root extract | Ingredient | Standard | Valid | Drug | RxNorm |
| DAHBJ | Cyperus rotundus root | 42898876 | 1307777 | Cyperus rotundus root extract | Ingredient | Standard | Valid | Drug | RxNorm |
| DACCL | Atractylodes lancea root | 42898612 | 1307719 | Atractylodes lancea root extract | Ingredient | Standard | Valid | Drug | RxNorm |
| JTS0001 | Individualized prescription [Decoction] | 900000020 | KIOM20 | Other decoction (personal prescription) | Clinical Drug Form | Standard | Valid | Drug | KIOM |
| JTS0001 | Gamidaebotang (Excellent empirical prescription) [Decoction] | 900000021 | KIOM21 | Gamidaebotang decoction | Clinical Drug Form | Standard | Valid | Drug | KIOM |
| DC341 | (DC) Jihaesocheonglyongtang (Granules) | 900000022 | KIOM22 | Jihaesocheonglyongtang granules | Clinical Drug Form | Standard | Valid | Drug | KIOM |
| JTS0001 | Sungihwalhyeoltang (Cheongganguigam) [Decoction] | 900000023 | KIOM23 | Sungihwalhyeoltang decoction (cheongganguigam) | Clinical Drug Form | Standard | Valid | Drug | KIOM |
| JTS0001 | Sopungtang-gami [Decoction] | 900000024 | KIOM24 | Sopungtang-gami decoction | Clinical Drug Form | Standard | Valid | Drug | KIOM |
| HJ73 | Hyanggisujoggagtang (Aromatic foot bath) therapy | 900000025 | KIOM25 | Hyanggisujoggagtang decoction | Clinical Drug Form | Standard | Valid | Drug | KIOM |
| DB046 | (DB) Cheongsanggyeontongtang (Granules) | 900000026 | KIOM26 | Cheongsanggyeontongtang granules | Clinical Drug Form | Standard | Valid | Drug | KIOM |
| JTS0001 | Oyagsungisan (Donguibogam) [Decoction] | 900000027 | KIOM27 | Oyagsungisan decoction (Donguibogam) | Clinical Drug Form | Standard | Valid | Drug | KIOM |
| JTS0001 | Banhabaegchulcheonmatang [Decoction] | 900000028 | KIOM28 | Banhabaegchulcheonmatang decoction | Clinical Drug Form | Standard | Valid | Drug | KIOM |
| DC341 | (Tsu) Jihaesocheonglyongtang (3g Granules) | 900000029 | KIOM29 | Jihaesocheonglyongtang 3g granules | Clinical Drug Form | Standard | Valid | Drug | KIOM |
| DGB17 | Boyanghwanotang [Decoction] | 900000030 | KIOM30 | Boyanghwanotang decoction | Clinical Drug Form | Standard | Valid | Drug | KIOM |
| DB039 | (DB) Ijungtang (Granules) | 900000031 | KIOM31 | Ijungtang granules | Clinical Drug Form | Standard | Valid | Drug | KIOM |
| JTS0001 | Jihwangeumja (Donguibogam) [Decoction] | 900000032 | KIOM32 | Jihwangeumja decoction (Donguibogam) | Clinical Drug Form | Standard | Valid | Drug | KIOM |
| JTS0001 | Sopungtang (Donguibogam) [Decoction] | 900000033 | KIOM33 | Sopungtang decoction (Donguibogam) | Clinical Drug Form | Standard | Valid | Drug | KIOM |
| JTS0001 | Geumsuyuggunjeon (Bangyaghabpyeon) [Decoction] | 900000034 | KIOM34 | Geumsuyuggunjeon decoction (Bangyaghabpyeon) | Clinical Drug Form | Standard | Valid | Drug | KIOM |
| JTS0001 | Cheongsimyeonjaeum (Taepyeonghyeminhwajegugbang) [Decoction] | 900000035 | KIOM35 | Cheongsimyeonjaeum decoction (Taepyeonghyeminhwajegugbang) | Clinical Drug Form | Standard | Valid | Drug | KIOM |
| DGB43 | Hwallaghyolyeongdan [Decoction] | 900000036 | KIOM36 | Hwallaghyolyeongdan decoction | Clinical Drug Form | Standard | Valid | Drug | KIOM |
| JTS0001 | Gamiondamtang (Bangyaghabpyeon) [Decoction] | 900000037 | KIOM37 | Gamiondamtang decoction (Bangyaghabpyeon) | Clinical Drug Form | Standard | Valid | Drug | KIOM |
| JTS0001 | Ojeogsaneohyeolbang (Bangyaghabpyeon) [Decoction] | 900000038 | KIOM38 | Ojeogsaneohyeolbang decoction (Bangyaghabpyeon) | Clinical Drug Form | Standard | Valid | Drug | KIOM |
| JTS0001 | Banhabaegchulcheonmatang (Bangyaghabpyeon) [Decoction] | 900000039 | KIOM39 | Banhabaegchulcheonmatang decoction (Bangyaghabpyeon) | Clinical Drug Form | Standard | Valid | Drug | KIOM |
| 137801ATB | Colchine( Colchicine 0.6mg) | 1101556 | 197541 | colchicine 0.6 MG Oral Tablet | Clinical Drug | Standard | Valid | Drug | RxNorm |
| 105001ATB | Allopurinol(allopurinol 100mg) | 1167323 | 197319 | allopurinol 100 MG Oral Tablet | Clinical Drug | Standard | Valid | Drug | RxNorm |
| 220101ATB | Antiroid(Propylthiouracil 50mg) | 1554073 | 198175 | propylthiouracil 50 MG Oral Tablet | Clinical Drug | Standard | Valid | Drug | RxNorm |
| 183601ATB | Synthyroid(Levothyroxine sodium 0.1mg) | 40169766 | 892246 | levothyroxine sodium 0.1 MG Oral Tablet | Clinical Drug | Standard | Valid | Drug | RxNorm |
| 249105ATB | Warfarin(warfarin sodium) | 40163554 | 855332 | warfarin sodium 5 MG Oral Tablet | Clinical Drug | Standard | Valid | Drug | RxNorm |
| 180101ATB | Matigen(ketotifen fumarate 1.38mg) | 986118 | 246127 | ketotifen 1 MG Oral Tablet | Clinical Drug | Standard | Valid | Drug | RxNorm |
| 225710CSI | Menocal nasal spray(salcatonin 1100IU) | 2030037 | OMOP4928787 | 1.4 ML salmon calcitonin 1100 IU/ML Nasal Spray | Quant Clinical Drug | Standard | Valid | Drug | RxNorm Extension |
| 194901ACH | Lipidil( micronized fenofibrate 200mg) | 19077245 | 310289 | fenofibrate 200 MG Oral Capsule | Clinical Drug | Standard | Valid | Drug | RxNorm |
| 221603ATB | Pyridoxin( pyridoxine HCl 50mg) | 44045292 | OMOP1039923 | pyridoxine 50 MG Oral Tablet | Clinical Drug | Standard | Valid | Drug | RxNorm Extension |
| 204401ACE | Ramezol( omeprazole 20mg ) | 43769269 | OMOP900554 | Omeprazole 20 MG Oral Capsule | Clinical Drug | Standard | Valid | Drug | RxNorm Extension |
